# Climate change and neurotropic vector-borne viruses: addressing emerging threats through a One Health approach

**DOI:** 10.1128/mbio.00886-25

**Published:** 2025-09-22

**Authors:** Kamalika Roy, Rajyashree Basu, Anirban Basu

**Affiliations:** 1 Division of Cellular and Molecular Neuroscience, National Brain Research Centre 29050, Manesar, Haryana, India; Albert Einstein College of Medicine, Bronx, New York, USA

**Keywords:** neurotropic viruses, vector-borne diseases, climate change, emerging threats, One Health

## Abstract

Vector-borne diseases are mainly transmitted through the bites of infected arthropods. They are a major public health concern as they account for more than 700,000 deaths annually. Among many vector-borne pathogens, the neurotropic viruses have been contributing to the increased number of deaths across the globe due to severe neurological complications. Despite the advancement of vector control strategies, the prevalence and severity of neurotropic viral infections have not been alleviated till date. Anthropogenic activities cause persistent fluctuations in temperature and weather trends. This plays a major part in shaping the fate of transmission dynamics and pathogenesis of such diseases. Changes in climatic factors, such as global warming and delayed withdrawal of monsoon, have had huge impacts on stretching the window of disease transmission worldwide. The abundance, survival, feeding activity, and vectorial competence of the arthropods are expected to increase with rising temperatures. This review aims to discuss how climate change affects ecosystems, thereby influencing vectors and the associated neurotropic viruses. It also highlights the urgent need for the “One Health” strategy. It is a concept that recognizes that humans and animals do not exist in isolation and are part of a larger ecosystem where their activity and health are interconnected to one another. This holistic approach is essential in addressing the emerging threats posed by climate change, rising rates of infection, and epidemics across the globe.

## INTRODUCTION

The pathology of the central nervous system (CNS) is a public health concern, and the arboviral neurotropic infections are a major contributor to the problem. Neurotropic viruses are potentially capable of invading the CNS, manipulating various cell populations, and promoting neuropathogenesis (1). Among many, the viruses belonging to the family Flaviviridae, such as Japanese encephalitis virus (JEV), dengue virus (DENV), West Nile virus (WNV), Zika virus (ZIKV), and Rhabdoviridae, such as Chandipura virus (CHPV), have been contributing to the increasing numbers of deaths due to severe neurological complications (Table 1). These neurotropic viruses are mainly transmitted by the mosquito and sand fly vectors (2, 3). JEV, a principal causative agent of viral encephalitis, is transmitted by the bite of the Vishnui group of *Culex* mosquitoes like *Culex tritaeniorhynchus*, *Culex vishnui*, etc. (4). Despite the advancement of vector control strategies, the annual clinical incidences of JEV have not been alleviated below 100,000. Among the patients with JEV, the case-fatality rate can be as high as 30% (4). Approximately 3 billion people are exposed to the risk of JEV infections in the endemic countries of Southeast Asia and the Western Pacific Regions (4). Dengue fever is also raising concerns day by day due to an eightfold increase in dengue cases globally, with approximately 100–400 million active infections (5, 6). DENV, transmitted by the bites of female *Aedes aegypti* and *Aedes albopictus* mosquitoes (5), leads to a broad spectrum of neurological manifestations like encephalopathy, encephalitis, meningitis, extensive neuroinflammation, and neuronal cell damage (2). The severity and prevalence of dengue are more alarming, as nearly 96 million of the global dengue infections manifest clinically, posing major risks to almost 3.9 billion people worldwide (5). *Culex*-borne neuroinvasive WNV, which was initially thought to be endemic to West Asia, the Middle East, Africa, and Europe, has been causing recurrent and fatal outbreaks in the United States in recent times (7). Cases of *Aedes*-transmitted Zika virus infections still persist in 89 endemic countries, including America (8). CHPV infection, transmitted primarily by sand flies like *Phlebotomus papatasi*, *Phlebotomus argentipes*, and *Sergentomyia* sp., also fairly accounts for a significant proportion of acute encephalitis syndrome (AES) in an endemic country like India. Sixty-four cases of CHPV-mediated AES have been reported in India between early June and mid-August 2024 (9). India has seen the largest outbreak of CHPV in the past 20 years (9). The high case-fatality ratio (56%–75%) from CHPV infection is alarming (9) and needs global attention.

**TABLE 1:** Overview of the emerging neurotropic arboviruses

Neurotropic virus	Vector	Major hosts	Global occurrence	Severity/cases	Clinical symptoms
Japanese encephalitis virus	*Culex* mosquito species like *Culex tritaeniorhynchus,* *Culex vishnui* (4)	Pigs and wading birds (amplifying host) (4, 10); humans (dead-end hosts)	Eastern, southern, and southeastern Asia, the Torres Strait of northern Australia, and Papua New Guinea (11)	100,000 cases reported annually (4)	General symptoms include headache, high fever, and anorexia; gastrointestinal symptoms include nausea, vomiting, and diffuse abdominal pain; neurological symptoms include acute flaccid paralysis, seizures, cognitive impairment, behavioral abnormalities, deep coma, focal weakness, hypotonia, and raised intracranial pressure (12)
Dengue virus	*Aedes* mosquito species like *Aedes aegypti* and *Aedes albopictus* (5)	Humans and non-human primates (monkeys) (13)	The Americas, Africa, the Middle East, Asia, and the Pacific Islands (14)	6.5 million human cases in 2023 (15); 13 million human cases in North, Central, and South America and the Caribbean in 2024 (16)	High fever (40°C), nausea, vomiting, severe headache, muscle and joint pain, pain behind the eyes, swollen glands, and rash; in cases of severe infection, some symptoms appear after the fever has subsided: severe abdominal pain, persistent vomiting, bleeding nose or gums, fatigue, blood in vomit or stool, rapid breathing, increased thirst (5)
West Nile virus	*Culex* mosquito species such as *Culex pipiens, Culex tarsalis, Culex quinquefasciatus* (7, 17)	Birds (amplifying hosts) (18); humans and horses (dead-end hosts) (19)	Africa, India, the Middle East, southern and central Europe, and North America (11)	2,445 human cases reported in the USA, with 165 (6.8%) confirmed deaths in 2024 (17); a total of 59,141 cases reported in the USA from 1999 to 2023 (20)	Around 80% of infected people are asymptomatic, with almost 20% displaying mild febrile illness with symptoms such as headache, vomiting, diarrhea, rash, joint pain, or body ache; less than 1% who are infected develop a severe neuroinvasive illness affecting the central nervous system, such as encephalitis or meningitis; some symptoms of the severe illness are high fever, headache, neck stiffness, disorientation, stupor, tremors, convulsions, muscle weakness, numbness, vision loss, coma, and paralysis (21)
Zika virus	*Aedes* mosquito species (8) like *Ae. aegypti* (22)	Apes, monkeys, and humans (23)	Tropical and subtropical areas of Africa, Southern Asia, Western Pacific, and the Americas (24)	42,127 reported cases in 2024, and 37,659 cases in 2023 in the Americas, with the highest portion reported in Argentina, Brazil, Bolivia, Costa Rica, and Colombia; 151 reported cases in India in 2024; 742 reported cases in Thailand in 2023 (25)	Symptoms similar to dengue, i.e., fever, headache, joint pain, rash, muscle pain, and conjunctivitis (red eyes); other symptoms may include encephalitis, meningitis, myelitis, blood disorder leading to bleeding, bruising, or slow blood clotting; the disease is congenital and can cause some serious birth defects like microcephaly and other pregnancy problems, including fetal loss, preterm birth, and stillbirth; Zika infection is also known to cause Guillain-Barré syndrome (26)
Chandipura virus	Sand fly species like *Phlebotomus papatasi*, *Phlebotomus argentipes*, and *Sergentomyia* sp. (9)	Humans (27)	Predominantly reported in the Indian subcontinent, along with sporadic detections in Sri Lanka, Senegal, and Nigeria (27)	245 cases of acute encephalitis syndrome, including 82 deaths, reported in India in 2024, of which 64 cases were confirmed to be CHPV infection (9)	Fever, convulsions, altered sensorium, vomiting, headache, and diarrhea (27)

**Fig 1:**
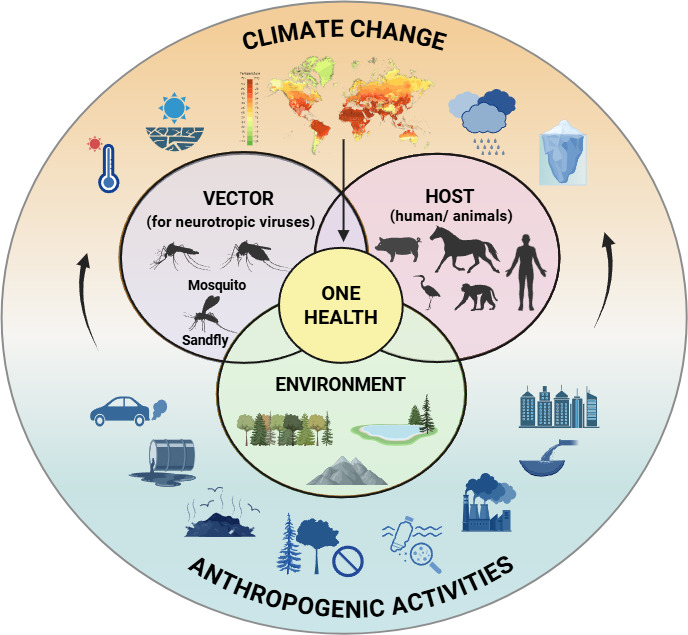
One Health recognizes that vector abundance, animal health, human health, and environmental parameters are interconnected to each other. The anthropogenic activities, such as deforestation, combustion of fossil fuels, oil spillage, urbanization, plastic pollution, etc., are causing drastic changes in the climatic variables like rainfall, temperature, and various other factors. The resultant dramatic climate shift is affecting One Health collectively by influencing the vectors, animals, humans, and environment. The image has been created with Biorender.com.

Several factors, like increasing urbanization, population growth, migratory populations, and changes in climatic factors such as global warming and delayed withdrawal of monsoon, have a huge impact on stretching the window of transmission of the neurotropic viruses across the globe (28). Climate can directly affect the disease-causing pathogen, the vector, the human and non-human hosts, and the environment (Fig. 1). These, in turn, determine the fate of the geographic outbreaks, transmission dynamics, emergence, and re-emergence of the neurotropic viruses (29).

Ambient temperature is critically important as the insect vectors carrying arboviruses are ectothermic and immensely susceptible to the slightest changes in temperature (29, 30). It determines the survival, feeding activity, and abundance of the vectors, as well as the rate of development of the pathogen within the vectors (28, 29). The extrinsic incubation period (EIP), the time between the intake of a pathogen by the vector through a blood meal from the infected host and the vector becoming infectious after the amplification and circulation of the pathogen inside the vector’s body, can be altered by temperature fluctuations (29, 31). The vector microbiome has a profound effect on the development of pathogens inside the vector. Climatic and environmental disruptions can also alter this microbial composition, thereby modulating the pathogen transmission (32). It has been reported that 49% of the emerging viruses are neurotropic, leading to encephalitis or other serious neurological clinical symptoms. More importantly, their emergences are triggered by environmental, ecological, or human demographic changes (33). Several reports have also suggested the expansion of brackish water bodies in coastal zones due to global warming-driven sea-level rise, followed by increased abundances of salinity-tolerant JEV-carrying mosquito vectors such as *Culex sitiens* and *C. tritaeniorhynchus*, as well as the adaptation of the DENV-carrying fresh-water mosquito vectors like *A. aegypti* and *A. albopictus* to salinity (34).

Therefore, the relationship between climate change, especially the warming of the earth due to anthropogenic activities, and the epidemiology of vector-borne diseases has grown a considerable amount of interest among researchers recently. However, there are contradictory reports on the correlation of ambient temperature with vector competence (30). A few studies have associated increased vectorial capacity with lower temperature (22, 35–37). On the other hand, a couple of studies on *Aedes* mosquitoes infected with DENV-2 (38, 39) and *Culex* mosquitoes infected with WNV (40) have shown that the rise in temperature is positively correlated with the infection rate and inversely correlated with the EIP. The current global expansion of some major arthropod vectors into temperate regions is adding to the complexity of this conflicting evidence (41). For example, the spread of *A. albopictus* from Southeast Asia to North America and Europe has led to the subsequent outbreaks of dengue fever in France and Hawaii (42–44). In the past years, DENV, WNV, and many other viruses have emerged and re-emerged in Asia, Europe, the Americas, and the Middle East (45). The Zika virus outbreak also caused an epidemic in South America (46). All of these neurotropic virus emergences are posing a threat to mankind and resulting in a global health crisis. Over the past few decades, consequences of the earth’s climatic shift have been drastically affecting the poor and developing countries that are already facing the worst infectious disease scenarios. The emergence of new transmission zones, even in the developed countries, is a matter of great concern. Since vector-borne diseases, including neurotropic viral ones, are influenced by an ecosystem that comprises a vector-pathogen-host tripartite relationship linked to environmental parameters, a multidisciplinary “One Health” approach is urgently needed to address this crisis. In this review, we focus on the current knowledge of the paradigm shifts in neurotropic arboviral transmissions driven by climatic deformations and a unified holistic approach as the method of prevention.

## IMPACT OF CLIMATE CHANGE ON THE VECTOR-BORNE NEUROTROPIC VIRAL INFECTIONS

Climate change is the persistent alteration in weather trends and temperature, which may arise naturally, like fluctuations in the solar cycle, or through anthropogenic activities. Since the 19th century, the primary cause of climate change has been mainly attributed to the combustion of non-renewable resources such as fossil fuels (like coal, oil, and gas) and other human activities (47). Climate change poses the most serious present-day challenge for humanity. The current unsustainable pattern of development is increasing the exposure of people and different ecosystems to climate hazards. The Intergovernmental Panel on Climate Change (IPCC) has reported that 3.3 billion of the world’s population is currently highly vulnerable to climate change, with the situation growing worse (48, 49). The rise of the mean global temperature by 1°C above preindustrial levels, due to anthropogenic activities, has had a profound impact on the climate (48). This includes an increase in the number of warm days and nights, extreme heat events, an accelerating rise in sea levels, and a decrease in snow cover. Extreme precipitation events have also been reported in both wet and dry regions (29). According to the IPCC in 2021, global temperature could rise by 2°C–4°C due to increasing emissions of carbon dioxide in the atmosphere. This rise is expected to be higher in the continental areas of the Northern Hemisphere (32). The poorest countries have been the worst hit and face the most due to the spread of infectious diseases caused by this climate crisis, the neurotropic arboviral infection being a prominent one among them (31, 49). It has been reported that climatic alterations can facilitate spillover between previously geographically isolated species. The global movement of pathogens and vectors and range shifts of wildlife will be amplified by climate, and these can put larger populations at higher risk of viral spillover events. For example, in the recent past, pandemics and epidemics like COVID, Ebola, and SARS have all been caused by viruses carried by rodents, bats, and other animals (50). The Zika virus, initially endemic in primates, began to be transmitted to humans by *A. aegypti,* which has now emerged in temperate countries due to climate change (51). Climate-induced biodiversity loss will also make ecosystems more susceptible to invasive species (50). Rapid thawing of the permafrost due to global warming increases the chances of novel disease outbreaks due to the release of unknown bacteria and viruses. Temperatures in the Arctic region have been reported to rise twice as fast as other regions across the globe, and two-thirds of the near-surface permafrost is projected to melt by 2100 (50). Scientists have predicted the emergence of new combinations of species at high elevations, in biodiversity hotspots, and areas with high human population density in Africa and Asia, which may enhance cross-species viral transmission by almost 4,000 times (52).

### Climate change and JEV infections

Studies from India, China, Vietnam, Taiwan, and Nepal have shown a significant association of temperature, rainfall, relative humidity, and solar radiation with JE cases (53). In the Gorakhpur district of India from 2001 to 2016, the use of generalized additive models to verify this association predicted that with every one-unit rise in mean temperature, relative humidity, and rainfall, the average JE admissions would increase by 22.23%, 5.22%, and 0.66%, respectively, and the JE mortality rate would increase by 13.27%, 3.27%, and 0.94%, respectively. In contrast, a decrease in wind speed and solar radiation by every unit increased JE admission by 11.42% and 17%, respectively, and JE mortality rate by 4.88% and 9.37%, respectively (53). The upsurge in JE cases has been linked to the increasing temperature and rainfall in China as well (54). Within a certain range, an increase in temperature results in the rapid development of larvae, shorter time between blood meals, and less incubation time for viral infection in mosquito vectors carrying JEV (55). Higher humidity allows longer survival and farther dispersal of mosquitoes. This allows them to feed on an infected animal and survive long enough to transmit the virus to humans or other animals (55). A study conducted in Chongqing in Southwest China showed that the number of JE cases began to increase when the temperature was greater than 16°C (55). In Taiwan, the number of JE cases increased when the temperature reached 22°C (56). Temperature has been a dominant factor influencing the monthly JEV outbreak in the Gansu Province of China (57). In India, the average temperature ranging between 22.8°C and 34.5°C facilitates JEV transmission (58), whereas the temperature ranging from 21.0°C to 25.2°C in China has been found optimal for the same (59). Thus, a JE outbreak may be facilitated by two factors: adaptations in agriculture (such as the use of pesticides, adoption of paddy cultivation, and creation of modern pig farms) and global warming. Temperature and precipitation have been reported to contribute to variations in the density of mosquitoes (59, 60) and, according to the Konno transmission model, are also highly related to the occurrence of JE (61, 62).

### Climate change and DENV infections

Around 40% of the world’s population is at higher risk of dengue fever as they live in tropical and subtropical climates (63, 64). It was initially thought that the disease was endemic in tropical regions; however, travelers in southern Europe also started getting infected (65). The northwest global expansion of dengue was driven by several factors, with climate change being the most crucial one among them (65). The *Aedes* mosquito population is blooming as cities expand, human population increases, and more people move to urban areas. This is especially true in overpopulated regions that have poor waste management and sanitation (66). *Aedes* mosquitoes may now spread the dengue virus for much longer into the year due to increased longevity and activity of the species at higher temperatures (66). Therefore, one profound effect of global warming is a longer transmission season. Clearly, climate change, caused by anthropogenic activities, is considered to be a major contributing factor in the rapid spread of this pandemic disease (63, 66), and it is estimated that around 60% of the world’s population will be affected by dengue fever by 2080 (63).

Dengue incidence has been shown to respond nonlinearly to temperature, peaking at 27.8°C and then declining at higher temperatures, with predicted maximum transmission at 29°C for *A. aegypti* and 26°C for *A. albopictus* (67). Some low-elevation equatorial areas are projected to see small declines in dengue incidence due to climate change. However, the majority of locations, including some cooler regions of Mexico, Peru, Brazil, Bolivia, and many of the largest cities in America, are predicted to see a 150% increase in incidence (68). Among the 21 countries included in a study, 15 countries were projected to see an increase under all scenarios (68). The National Center for Vector Borne Diseases Control (NCVBDC) states that dengue cases have quadrupled from 2015 to 2023 in India. The optimal temperature range for increased dengue transmission is a mean range of 27°C–35°C, although it mostly varies based on local climatic conditions (69). In Singapore, a 14% increase in the incidence rates of dengue within a 40-year span has been attributed to rising temperatures (70). In Brazil, the areas that were more urbanized and exposed to high temperatures for a prolonged time between 2014 and 2020 had higher incidence rates of dengue (71). On the other hand, the optimal temperature for disease propagation can vary depending on the local and regional climates, too. In India, the rise in temperature, even up to 39°C, has facilitated dengue transmission in Delhi, whereas temperatures ranging between 27°C and 35°C were optimal for the same in Pune (69). A study conducted in Pune between 2004 and 2015 has shown that moderate rainfall over a prolonged period can increase the dengue mortality rates, whereas heavy rains can reduce it by flushing out the vector’s habitat. A relative humidity of 60%–78% has also been reported to be optimal for dengue transmission in this region (69).

### Climate change and WNV infections

WNV has been experimentally shown to replicate across a broad range of temperatures, starting from 14°C in ectothermic mosquitoes (72) to 45°C in febrile avian hosts (73). The temperature threshold for the survival of *C. tarsalis* is demonstrated to be between 14°C and 35°C, where temperature positively correlates with the development of the vector (74, 75). The replication cycle was shown to be completed more quickly in mosquitoes at higher temperatures (76). A clear link was present between the intensity of the outbreak in humans and extreme heat (77, 78). However, extremely high temperatures have been shown to cause a decline in mosquito activities, including reducing larval survival and virus growth (79). Warmer conditions enable the spread of WNV in new areas by expanding the seasonal abundance and range of the vector species, as well as increasing their transmission competence (79). High spring and summer temperatures, drier winters, and water scarcity, along with changes in land, such as frequently irrigated crops and highly fragmented forests, were found to be the major determinants for increased WNV incidence across Europe (80). Researchers studied the association between heatwaves and WNV incidence in humans in Israel and showed that an extreme temperature rise early in the summer is a good indication of rising populations of vectors (81). Platonov et al. (82) studied the outbreaks in the Volgograd province in Russia and showed that years with milder winters and hotter summers led to an increase in *Culex* mosquitoes during the epidemic season. The year 1999 marked the first appearance of WNV in the Western Hemisphere, starting from New York City (83), then reaching Canada and Central America by 2002, thereafter spreading to the Pacific coast (California) by 2003 (84), and then to Argentina by 2005 (85, 86), where it infected numerous species of birds, along with humans and other mammals. It became endemic across most North American temperate regions (87), and the initial outbreak in the USA is thought to be due to a prior drought (87, 88). WNV is a notable concern in the Canadian Prairies, where it has been observed that warmer and drier summers have led to an increased scarcity in water levels (89, 90). Observations made in Florida note that spatial and temporal differences in periods of droughts and rain events are associated with the increase in WNV infection in humans and sentinel chickens (91–93). Drought conditions lead to an increase in the number of larval breeding sites with fewer competitors, as well as less mosquito predators, with closer proximity of birds and mosquito vectors that enhance virus transmission, hence increasing the prevalence of vector populations in semi-permanent wetlands. Springtime drought, followed by a wet summer, was reported to be a reliable predictor of the incidence of WNV infections in humans (91–93). WNV is known to affect multiple species of mosquitoes and birds and currently has a widespread distribution across Africa, southern and eastern Europe, western Asia, the Middle East, and Australia. Biodiversity loss is also thought to promote patterns of transmission of disease as vector-host encounter rate increases with the decrease in host community diversity (94). In Missouri, a negative correlation has been found between WNV infection in vectors and bird diversity at the regional scale, and at a national scale with humans in the USA (95). However, one report suggests that avian biodiversity loss can also lead to a decrease in mosquito infection rates and avian seroprevalence in Atlanta (96). In the USA alone, between 1999 and 2021, more than 55,000 cases of WNV have been reported, out of which more than 27,500 cases developed neuroinvasive disease and more than 2,500 deaths (80, 97).

There are cases of WNV emerging or re-emerging at the edges of current endemic zones and in high-latitude regions (98, 99). Similarly, by 2050 and 2080, the suitable range for WNV in North America is projected to extend northward and to higher altitudes, potentially leading to both native and non-native species getting infected (98). Expansion of outbreak locations and high-risk areas is predicted for the future in Europe and South America as well, with more pronounced changes expected under high greenhouse gas emission scenarios (100).

### Climate change and ZIKV infections

Since 2013, the Zika virus has spread to at least 49 countries and territories (101), reporting an estimated 150,000–500,000 cases, with Brazil alone facing 3,000 cases (102). There has been some research linking the 2015 Zika outbreak to El Niño in South America (103). Research done during the 2016 Zika outbreak suggests that transmission may be restricted to warmer and less seasonally variable parts of the world as compared to dengue (104), and the minimum temperature for ZIKV transmission by *A. aegypti* is around 5°C higher than that of DENV (105, 106). It is predicted that if climate change is not mitigated, as many as 1.33 billion new people (1.17 billion outside the Line of Actual Control or LAC) could be pushed into areas that would become suitable for ZIKV transmission with respect to temperature, even though it is only confined to the tropics currently (106). It is estimated that a total of 737 million people worldwide (635.8 million outside LAC) are at risk of exposure to year-round climate suitability that aids in ZIKV transmission, especially in South and East Asia, and sub-Saharan Africa (106). Some of the regions with populations of 100 million or more people, which are projected to experience higher rates of ZIKV transmission due to climate suitability, are East Africa, North Africa, high-income North America, East Asia, the Middle East, and Western Europe (considering regions designated by the Global Burden of Disease or GBD study) (106). Usually, a temperature ranging from 18°C to 34°C is ideal for ZIKV transmission, with a peak at 29°C (107). The warming of winter temperatures enhances the overwinter egg survival of *A. aegypti,* which in turn expands the geographic range of this vector mosquito (107). Similarly, higher temperatures in seasons like spring, summer, and autumn actually prolong the transmission season, even in temperate regions (107). Other than the temperature, the precipitation rate also influences the transmission dynamics of ZIKV. It has been reported that the ZIKV outbreak in America during 2015–2017 was inversely correlated with precipitation, whereas drought actually enhanced the viral transmission (106).

### Climate change and CHPV infections

While endemic to India, CHPV is also likely to be present in other countries in Asia and Africa (108). The virus has been reported to persist in parts of Gujarat, Maharashtra, and Telangana, and its recurring outbreaks have become a health concern, affecting mostly children below the age of 15 (108, 109). It is yet to be explained why these parts of India are affected by the CHPV virus despite Phlebotomine sand flies being prevalent in all parts of the country. The virus remains infective in sand flies and cell culture supernatant at 4°C for 8 weeks, and it remains viable at 37°C for 18 days in infected cell supernatants. However, the virus loses virulence within a week when infected sand flies are stored at 37°C (109). Climate change may expand the geographic footprint of epidemics caused by CHPV through shifts in temperature, humidity, and seasonal monsoon patterns. It can accelerate CHPV transmission into newer regions, thus amplifying the risk of widespread and more frequent epidemic outbreaks (110). Despite the decrease in the case numbers of CHPV-mediated AES, further outbreaks can be triggered by climate change and drawn-out monsoons (108).

## ONE HEALTH STRATEGY TO COMBAT THE CRISIS

The alarming expansion of the geographic ranges of neurotropic arboviral infections caused by JEV, DENV, WNV, Zika, or CHPV is triggered by certain environmental fluctuations. These fluctuations affect three main components: the virus, the arthropod vector, and the vertebrate host. The transmission of the neurotropic arboviruses happens through the following cycles, such as the enzootic or sylvatic cycle, the human-amplified or urban cycle, and the epizootic or rural cycle (111). In the sylvatic cycle, the virus is maintained in the ecosystem through interactions between the birds or wild animals, such as non-human primates, rodents, etc., and the associated arthropod vectors (23). For many neurotropic arboviruses, there are dead-end hosts like domestic animals or humans. These hosts can stop the transmission cycle of the virus as the viral load in their blood is too low to be transmitted by the biting vectors, but the clinical symptoms of the disease can still persist in them (112). For example, the transmission of JEV and WNV involves sylvatic cycles where the vector *Culex* mosquitoes spread the viruses among birds, which are the amplifying hosts. They can harbor the viruses in them for several days, and their migration can carry the viruses over long distances (113). Pigs also act as the amplifying hosts for JEV (113). The vector-borne transmission of both of these viruses infects various other vertebrates, including dead-end hosts such as humans and equines (113). Rhabdoviruses are also known to infect a wide range of hosts, such as vertebrates (like mammals, fish, birds, and reptiles), invertebrates, protozoans, plants, and fungi. One such virus is CHPV. The transmission cycle of CHPV in nature involves sand flies, rodents, and small mammals (114) and has been isolated from hedgehogs and sand flies in Nigeria and Senegal (115, 116). It has also been isolated from humans (117–119). Anti-CHPV antibodies have been found in humans (117, 119, 120), toque macaques (121), and cattle, pigs, goats, sheep, and other domestic animals (122), and a broader geographical distribution was indicated when antibodies against CHPV were reportedly found in monkeys in Sri Lanka (116). Infected domestic animals only developed ulcers at the inoculation site, and no other clinical symptoms were shown in experiments. Isolating the virus from tissues other than the inoculation site was unsuccessful. Thus, further research about the disease and how animals respond to it under natural conditions is lacking (114). Contrastingly, several other neurotropic arboviruses can be transmitted among humans by vector bites. Viruses like Zika and DENV were initially transmitted by forest mosquitoes among the African and Asian primates, respectively. However, the vectorial capacity of the anthropophilic *A. aegypti* mosquitoes started the urban cycle of these viral transmissions, making humans a new host (123, 124). Therefore, entry of a virus from the sylvatic to the urban cycle can result in the rapid spread of the virus by anthropophilic mosquitoes. On the other hand, the rural cycle involves domestic animals serving as amplifying hosts, leading to the enhanced spillover of viruses to humans in agricultural settings (125). In the context of human medicine, the urban cycle is of utmost importance because the widespread urban population is prone to massive propagation of the infection. Similarly, the areas with dense livestock populations are at risk of the rural cycle of viral transmission. So, the viruses that follow these cycles should be given attention from a consolidated perspective. To successfully restrict the emergence of such viruses by a One Health approach, surveillance is the foremost step, which will help to monitor the viral spread, identify livestock and humans at risk, introduce vaccination programs in areas with high rates of viral transmission, and ultimately offer means to control the disease (126). In new areas, which were previously free from emerging viral threats, the increased prevalence of vector-borne neurotropic viral infections is being facilitated by climatic alterations such as changes in average rainfall, flood situations, and rising temperatures. An integrated approach is always welcome to understand the interconnectivity between the climate, zoonoses, and public health and mitigate the dramatic resurgences of these viruses.

### One Health approach to control JEV transmission

Determining the abundance and seasonal prevalence of vector mosquitoes and their infection rates can indicate the possibility of JE outbreaks in any particular region. The distribution of the vectors is reported to be proportionally related to the JE outbreaks in India and Korea (127–129). Weather events can also regulate the population dynamics of the vectors, thereby being potential predictors in forecasting JE upsurges. Vector surveillance has been given more importance as a tool to monitor JEV emergence in Australia (130). The genetic heterogeneity among the JEV isolates and re-emergence of JEV in the temperate regions can be attributed to the migratory viremic birds as well. Migratory birds from China were reported to result in the shift in the widespread genetic variant of JEV in Japan, Korea, and Vietnam (126, 131). Therefore, serological surveys among sentinel birds and wild or domestic animals may be useful in forecasting JE epidemics. In Australia, routine mosquito and reservoir host surveillance, prior to the JEV transmission to humans, has provided early warnings about the extent of the disease outbreak (132). Surveillance is followed by control measures like mass vaccination among sentinel animals and humans. It is thought to be effective as the number of JE cases in horses, pigs, and humans has been reduced with the advancement in the production and scale-up of JE vaccines (126). However, the cost, requirement of booster doses, and long-term follow-up to maintain immunity, along with a few reported adverse side effects, including allergic reactions, unavailability of the vaccination program during severe outbreaks, especially in poor or developing countries, are the major constraints (126, 133). Hence, the non-vaccine control has to play an equally important role here. The changes in land cover and use, followed by the environmental changes in habitats due to extensive anthropogenic activities, are shaping the risk pattern of humans getting infected with JEV (134). The tropical regions having a large cover of rice fields are more prone to JEV dissemination (135). These rice fields offer excellent breeding sites for *Culex* mosquitoes and foraging and resting sites for birds (134). Moreover, increased domestic and industrial pig farming is providing a potential blood meal source for the mosquitoes. So, rearing pigs away from humans, the use of barn fans to protect animals from mosquito bites, and biocontrol of the JEV-carrying mosquitoes by using plant-based insecticides or larvivorous fishes may presumably hamper the transmission dynamics of JEV (126). Intermittent drainage of the rice fields can also limit the vector prevalence and subsequent disease occurrences (126, 136). Besides the vaccination strategy, the implementation of modern agricultural practices and moving human populations away from the animal farms were crucial factors that facilitated the drastic reduction in JE cases in an endemic country like Japan (126). In the Gorakhpur district of India, where several climatic factors like excessive rainfall, flood situations, cultivation of paddy, low topography, and accumulation of silt in riverbeds favored mosquito breeding leading to the JEV transmission, a multidisciplinary One Health strategy had been able to limit the disease burden by reducing the morbidity and mortality rate by 90% (137). Thus, elaborate research on the various aspects of the JEV transmission cycle is required to eradicate the chances of sudden and rapid outbreaks.

### One Health approach to control DENV transmission

The severe impact of climate change on dengue transmission, the global threat it poses, and fragile vaccination strategies emphasize the requirement of a unified One Health strategy for disease management. In addition to the vector control programs, recent reports suggest that the application of omics science and biotechnology to identify new vaccine candidates and prophylactic approaches to counter the viral threat (138). Dengue control program in China, by implementation of the One Health strategy, can be an excellent case study for its efficacy in minimizing disease fatalities (139). The rise in density of *A. aegypti* and *A. albopictus* populations due to increased precipitation and temperature in China led to dengue outbreaks that could only be handled by multi-sectoral interventions. China’s swift response in real-time data collection and analysis of the dengue cases at the onset of outbreaks offers a compelling framework that can be adapted by the dengue-endemic countries. In 2018, the monitoring of meteorological updates, vector density, and study of the case numbers helped predict and prevent a potential dengue outbreak in Guangdong Province (139, 140). Vector surveillance in 23 Chinese provinces identified the prevalence of *Aedes* mosquitoes. In addition, sentinel surveillance, serological monitoring, and epidemiological investigative procedures across 16 districts of Hainan, Guangdong, Yunnan, Fujian, etc., have been proven fruitful in resisting this public health challenge (139, 141). The collaboration between Yunnan Province and its neighboring Southeast Asian countries, through data sharing, enhances the chances of regional prevention and restricts cross-border dengue transmission (142). Also, the *Wolbachia*-based population replacement of *Aedes* mosquitoes by the successful release of mosquitoes having novel *Wolbachia* infections is now being carried out in China to effectively control the vector-borne DENV spread by biological means (143–145). Thus, China has achieved a significant decline in dengue cases (19,451 cases) in 2023 compared to the previous five years (43,095 cases) (139). Moreover, certain provinces like Guangdong and Yunnan have reported remarkably lower incidence rates in comparison to their neighboring countries like Sri Lanka, Malaysia, and Laos in 2023 (146, 147). Through the effective application of an integrated One Health program, China has been able to reduce the mortality rates due to dengue fever to 0.01 per 100,000 between 2005 and 2023 (139). The One Health program is being adopted as a tool to fight dengue and other vector-borne zoonotic diseases not only in China but also in Brazil (148).

### One Health approach to control WNV transmission

Studies in the recent past have taken genomic surveillance of the propagating WNV into account to develop the framework of One Health surveillance. This strategy has helped in acquiring detailed knowledge of the viral evolution pattern and the influence of viral genetics on the morbidity and mortality rates of West Nile fever in the endemic regions of Italy during 2023 (149). Whole-genome sequencing, along with the epidemiology and clinical diagnosis, aided in characterizing the indigenous and imported WNV circulating at the vector-animal-human interface in Romagna (149). In the WNV-endemic Veneto region of northeastern Italy, a One Health-based surveillance plan effectively brought down the WNV infection rates among mosquitoes and birds within a year. The active human WNV infections had also been reduced to 56 in 2023 from 531 in 2022 (150). All these findings collectively show a way forward to eliminate WNV.

### Predictive models for global projections of the neurotropic viral diseases under changing climate trends

Various observational and experimental studies can be performed to understand the climate-disease linkage. Observational studies include retrospective and prospective analysis of the fluctuations in climate variables and disease patterns, along with interregional comparisons. Experimental studies range from laboratory-based reductionist studies at the molecular and organism levels to field-based manipulation studies at the population level (151). There are certain analytical and advanced modeling approaches that offer quantitative global projections of disease transmission risk under various climate change scenarios. Mechanistic or process-based models use the theoretical knowledge of the biophysical mechanisms to simulate the health impacts of climate change, whereas empirical-statistical models use the empirical data on the past trend of variations to predict the future pattern of changes in the studied variables (151). A detailed dynamic simulation model of the abundance of vector mosquitoes such as *Anopheles*, *Aedes*, and *Culex* (91), or a matrix population model of the dynamics of *Culex quinquefasciatus* under fluctuating weather trends, made valuable contributions to the field (152). Predictive models were also used to project JEV outbreaks in Australia (153) and China (154). A joint spatiotemporal modeling revealed the seasonal variations in the abundance of JEV vectors across India (155). The maximum entropy model projected the distribution of *A. aegypti* and *A. albopictus* across mainland China for 2041–2100, based on the impact of annual mean temperature, seasonality of temperature, and precipitation. The results predicted the expansion and emergence of suitable habitats for both species of mosquitoes (156). A predictive model based on the temperature and precipitation data of Reunion Island in France showed that decreasing precipitation is going to negatively impact the *A. albopictus* abundance in low-elevation areas. Contrarily, at mid and high elevations, a significant warming will counterbalance the decreasing precipitation, leading to increased abundance of this dengue vector in 2070–2100 (70). Interestingly, the different thermal niches of these two dengue vectors, *A. aegypti* and *A. albopictus*, show different shift patterns under climatic variations. The temperature-dependent, month-wise global transmission risk by these vectors was predicted by using a model of viral transmission that was empirically parameterized. The prediction was also compared with the projected risk in 2050 and 2080 based on general circulation models. The results predicted that severe climate change scenarios will expose a larger population to transmission by heat-tolerant *A. aegypti*, not by heat-limited *A. albopictus*. In South Asia and sub-Saharan Africa, the transmission potential of *A. aegypti* throughout the year is likely to expand, whereas the transmission potential of *A. albopictus* is expected to decrease, as a result of tropical warming (157). On the other hand, a few reports suggest that *A. albopictus* is a more invasive vector than *A. aegypti*, as it has a greater niche and range expansion over its shorter invasion history. Therefore, it is important to pay more attention to *A. albopictus* if the future climate changes promote their invasiveness (158). Another model also predicted that *A. albopictus* may have a greater capacity to transmit DENV than *A. aegypti* due to temperature and other contributing factors (13). In the recent past, a group of researchers assessed the risk of WNV outbreaks in Europe using an ensemble climate model and a multi-scenario approach. The projections of area-specific outbreaks and populations at risk were estimated, which predicted up to a fivefold increase in WNV infections in Europe by 2040–2060, due to the climatic alterations, compared to 2000–2020. The WNV-affected land areas could increase from 15% to 23%–30%, putting 161 to 244 million European people at risk (159). Predictive spatial models were also used to estimate the risk of WNV exposure in Colorado (160). In Eastern Croatia and Northwestern and Northeastern Turkey, the climate change predictions for 2025 revealed a higher probability of WNV infection, which is likely to expand more in 2050, along with a significant increase in the prevalence of infection in blood donor populations in the WNV-affected areas (99). Therefore, such modeling-based projections can draw attention to targeted public health responses to manage the crises.

### Gaps and challenges

In recent times, increasing numbers of imported cases and severe epidemics across the globe have intensified the magnitude of the challenges faced in this domain. For example, at least 10 million cases and 6,000 deaths due to dengue have been reported in more than 80 countries as of 2023 (139). This global surge is a major factor behind the increase in the number of non-native dengue cases, even in a country like China, where the dengue control program has been proven effective (139). Population movement and urbanization are also impacting the neurotropic virus transmission and vector proliferation, respectively. In Malaysia, the dengue case numbers are much higher in residential urban areas than in industrial or commercial zones due to the urbanization-mediated changes in patterns of land use (139). Several preventive measures are falling short, too, such as periodic irrigation of the paddy fields to limit JEV transmission, which can be impractical in many rural areas (126). During vector monitoring, the requirement of skilled and adequate manpower for operating the equipment and identifying the species is often not meeting the standard. The emergence of insecticide-resistant vector species is another serious concern. In addition, the lack of effective therapeutics or vaccines, impaired public awareness, and inadequate community participation are hurdles that need to be overcome.

### Road ahead

Despite the existing challenges, certain agendas such as improving the risk assessment team’s early detection abilities, organizing rapid response teams, deploying modern information technologies, clarifying and distributing tasks and responsibilities among multiple sectors, sufficient information sharing, advancement of diagnostic techniques and eco-friendly control strategies, strengthening collaborations, focusing on the translational research, elimination of the vector breeding sites, betterment of the community sanitation, environmental governance, infrastructural development, government funding, and public health awareness campaigns can be adopted for the abatement of the crisis. Joint ventures of several sectors and disciplines are progressing toward the elimination or at least reduction of vector-borne neurotropic viral infections caused by JEV, WNV, or DENV in endemic countries all over the world. The model can also be executed in India to diminish fatal CHPV outbreaks. To ensure optimal protection from Zika fever, endemic countries like Singapore are also relying on the One Health concept (161).

## CONCLUDING REMARKS

This study systematically summarizes the potential threat of rapid climatic alterations, their profound implications on transmission dynamics, and the unprecedented geographical spread of neurotropic arboviruses. It reviews a comprehensive One Health approach that works at the regional, national, and global levels to obliterate the mentioned threat. As the exposure to these viruses leads to a wide spectrum of deleterious effects on human health, the progress toward restraining the viral propagation needs to be escalated, and this goal can only be achieved by integrated surveillance, interdisciplinary research, and intersectoral coordination.
